# Supplementary material for: Overexpression of 9-cis-Epoxycarotenoid Dioxygenase Gene, IbNCED1, Negatively Regulates Plant Height in Transgenic Sweet Potato
Source: Int J Mol Sci. 2023 Jun 21;24(13):10421. doi: 10.3390/ijms241310421 (PMC10342018; doi:10.3390/ijms241310421)
Supplement: Supplementary file 1 [file ijms-24-10421-s001.zip › ijms-2353208-supplementary.docx]

Article

Overexpression of 9-*cis*-Epoxycarotenoid Dioxygenase Gene, *IbNCED1*, Negatively Regulates Plant Height in Transgenic Sweet Potato

| **Citation:** Zhou, Y.; Zhao, C.; Du, T.; Li, A.; Qin, Z.; Zhang, L.; Dong, S.; Wang, Q.; Hou, F. Overexpression of 9-*cis*-Epoxycarotenoid Dioxygenase Gene, *IbNCED1*, Negatively  Regulates Plant Height in  Transgenic Sweet Potato.  *Int. J. Mol. Sci.* **2023**, *24*, x. https://doi.org/10.3390/xxxxx  Academic Editor: Tomotsugu Koyama  Received: 3 April 2023  Revised: 8 June 2023  Accepted: 16 June 2023  Published: date  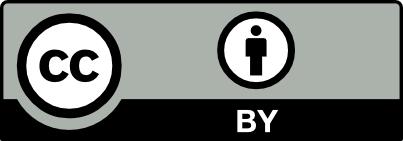  **Copyright:** © 2023 by the authors. Submitted for possible open access publication under the terms and conditions of the Creative Commons Attribution (CC BY) license (https://creativecommons.org/licenses/by/4.0/). |
| --- |

Yuanyuan Zhou, Chunling Zhao, Taifeng Du, Aixian Li, Zhen Qin, Liming Zhang, Shunxu Dong, Qingmei Wang* and Fuyun Hou*

Crop Research Institute, Shandong Academy of Agricultural Sciences, Jinan 250100, China;
zhou_yy_2020@163.com (Y.Z.); mm13573946870@163.com (C.Z.); a986947745@126.com (T.D.); 13688628863@163.com (A.L.); qin_zhen2001@163.com (Z.Q.); zhanglm11@sina.com (L.Z.);
dsxu16@163.com (S.D.)

***** Correspondence: wangqingmei@shandong.cn (Q.W.); houfuyun@shandong.cn (F.H.)

Supplementary Materials

**Figure S1.** The results of reads mapping (**A**, **B**), the correlation analysis of six samples (**C**), the PCA analysis of six samples (**D**), and the differentially expressed genes between two parents (**E**).


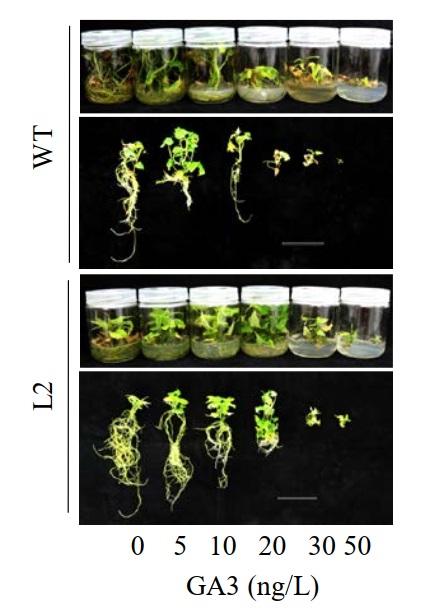


**Figure S2.** Phenotypes of *in vitro*-grown transgenic sweet potato plants and WT cultured on MS medium with 0, 5, 10, 20, 30 and 50 ng/L GA3 for 4 weeks.

**Table S1.**The quality summary of RNA-Seq data.

| **Sample** | **Raw Reads** | **Raw Bases(G)** | **Clean Reads** | **Clean Base(G)** | **Error Rate(%)** | **Q20(%)** | **Q30(%)** | **GC Content(%)** | **Reads mapped** |
| --- | --- | --- | --- | --- | --- | --- | --- | --- | --- |
| CK-1 | 107174170 | 16.08 | 101388086 | 15.21 | 0.03 | 97.71 | 93.35 | 46.52 | 79452424(78.36%) |
| CK-2 | 110935456 | 16.64 | 107139790 | 16.07 | 0.03 | 97.48 | 93.62 | 46.87 | 85038225(79.37%) |
| CK-3 | 102752088 | 15.41 | 100329656 | 15.05 | 0.03 | 97.56 | 92.93 | 45.57 | 79037922(78.78%) |
| OE-1 | 117839154 | 17.67 | 115148484 | 17.27 | 0.03 | 97.64 | 93.33 | 45.86 | 90357126(78.47%) |
| OE-2 | 102118192 | 15.32 | 100612266 | 15.09 | 0.03 | 97.69 | 93.21 | 45.48 | 79744707(79.26%) |
| OE3 | 90922168 | 13.64 | 89665004 | 13.45 | 0.03 | 97.33 | 92.46 | 45.32 | 70566702(78.70%) |

**Table S2.**Primers used in this study.

| **Primer name** | **Primer sequence (5’-3’)** | **Application** |
| --- | --- | --- |
| *IbNCED1*-F | GGTACCATGGCCAACACCATT | vector construction |
| *IbNCED1*-R | GTCGACAGCTTGGGTGGATAG | vector construction and gDNA PCR |
| 35S-F | GACGCACAATCCCACTATCC | gDNA PCR |
| *Ibactin*-F | AGCAGCATGAAGATTAAGGTTGTAGCAC | qRT-PCR |
| *Ibactin*-R | TGGAAAATTAGAAGCACTTCCTGTGAAC | qRT-PCR |
| *IbNCED1*-qF | ATTCCCACTTCAATATCCACTGCC | qRT-PCR |
| *IbNCED1*-qR | TTGCCGCCGCTCTTTGC | qRT-PCR |
